# The silent epidemic of non-adherence – insights from the 2024 a:care congress

**DOI:** 10.1186/s12919-025-00326-4

**Published:** 2025-05-22

**Authors:** Sheri D. Pruitt, Rabia Khan, Nathorn Chaiyakunapruk, Arintaya Phrommintikul, Miguel Angel Díaz Aguilera, Ngiap Chuan Tan, Shoaib Afzal, Andressa da Silva van der Laan, John Weinman

**Affiliations:** 1 Former Director of Behavioral Science Integration, Independent Behavioral Science Consultant, Kaiser Permanente, Ada, OK USA; 2 School of Population Health, University of New South Wales, New South Wales, Australia; 3 Department of Pharmacotherapy, College of Pharmacy, University of Utah, Salt Lake City, Utah, USA; 4 Division of Cardiology, Department of Internal Medicine, Faculty of Medicine, Chiang Mai University, Chiang Mai, Thailand; 5 National Center for Preventive Programs and Disease Control, Ministry of Health, Mexico City, Mexico; 6 SingHealth Polyclinics, Singapore, Singapore; 7 Duke NUS Medical School, Singapore, Singapore; 8 Department of Clinical Biochemistry, Copenhagen University Hospital Herlev and Gentofte, Gentofte, Denmark; 9 Global Innovation and Development, Abbott Product Operations AG, Allshwil, Switzerland; 10 Institute of Pharmaceutical Science, King’s College London, London, UK

**Keywords:** Medication adherence, Non-adherence, Congress, Behavioral science, Behavior patient-physician relationships, Health economics, Digital health technologies, Adherence stakeholders

## Abstract

Medication non-adherence is a global challenge with profound implications for patient health outcomes, healthcare systems, and society at large. This widespread issue leads to hundreds of thousands of preventable deaths and hospitalizations annually, and billions of dollars in avoidable healthcare costs. No country is immune to this challenge. The a:care Congress 2024, titled "Adherence is a Behavior: Do We Have the Right One?", addressed this critical issue to understand its societal and economic burden and explore the complex human behaviors driving it. The Congress examined the global impact of non-adherence and local realities, providing a health economic perspective on its consequences. Behavioral factors influencing adherence were analyzed, emphasizing the importance of understanding individual patient motivations. The role of technology and artificial intelligence in the physician–patient relationship was discussed, highlighting opportunities and challenges of integrating these tools. The patient's voice was brought to the forefront, underscoring the importance of communication and trust in the care relationship. A final multidisciplinary session explored the role of each stakeholder in improving adherence. This article provides an overview of the different sessions from the congress, focusing on the challenges associated with medication non-adherence and potential solutions. It emphasizes the need for a multidimensional approach involving all healthcare system stakeholders and the importance of prioritizing the development of new strategies to improve overall health outcomes and healthcare system efficiency.

## Background to the a:care congress 2024

Prescription medications are used to prevent, treat or relieve symptoms of a disease or abnormal condition [1]. However, the effectiveness of an intervention is dependent on patients adhering to prescribed regimens. A report from the World Health Organization (WHO) revealed that nearly 50% of patients do not take their medications as prescribed, with approximately 30% failing to fill their first prescription [2]. This widespread issue of non-adherence poses a significant public health challenge, leading to adverse health outcomes, including nearly 200 000 preventable deaths and hospitalizations annually, and billions of dollars in avoidable healthcare costs [3].

In light of this global challenge, the *a:care* program was created in 2021. This initiative leverages behavioral science to explore the complexities of non-adherence, including its underlying mechanisms, contributing factors, and diverse root causes. The program aims to raise awareness of the burden posed by non-adherence, advocate for improved adherence strategies, and elucidate the roles and responsibilities of stakeholders involved in addressing this issue. One of the key initiatives within the a:care program is the a:care Congress. Two prior editions of the Congress were held in 2021 and 2022, with the respective themes: *Medication Adherence: What Can You Do with the Medical Challenge That Starts After the Consultation?* and *Adherence to Treatment: Global Challenge, Tangible Solutions.* Ten thousand and 15,000 attendees from 107 countries were connected, respectively. The third a:care Congress, titled *Adherence is a Behavior: Do We Have the Right One?*, focused on understanding the societal and economic burden of non-adherence and the intricate human behaviors driving it. Physicians, patients, epidemiologists, health economists, medical societies, and behavioral science experts convened to share insights and discuss innovative, multidisciplinary strategies to tackle this critical healthcare challenge.

As part of the virtual registration for the 2024 edition of the a:care Congress, participants were invited to complete an optional survey, which garnered responses from 4,464 individuals. Among them, 212 participants attended both the first and second editions, 421 attended only the first edition, and 156 only the second. A total of 3,441 participants attended the congress for the first time. The results revealed notable insights: among Health Care Providers (HCPs) who participated in at least one a:care Congress in 2021 or 2022, 50% reported a strong understanding of the drivers of non-adherence, compared to only 18% in the group that attended the Congress for the first time. 31% of these HCPs found it very easy to assess the risk of non-adherence, significantly higher than the 7% in the first-time group. The survey also revealed that 84% of a:care HCPs have implemented behavioral change techniques in their practice, compared to 60% in the first-time group, while 77% used tools to measure patient adherence, compared to 47% in the group that attended for the first time. These findings highlight the role of the a:care program in raising HCPs’ awareness of non-adherence and enhancing their capacity to address and manage patient adherence.

In 2024, the Congress was a four-hour virtual event broadcasted globally (one session live and one session delay depending on the time zone) connecting 17,804 HCPs in total. The speakers convened in Chicago, Illinois, USA, delivering their presentations from a centralized location. Each session was followed by a live Q&A segment, during which the audience submitted questions via a chat interface, enabling the moderator to relay these inquiries directly to the speakers. The program featured a series of six sessions, beginning with an opening address by Sheri D. Pruitt, Ph.D, John Weinman, Ph.D, and Ms. Kathryn McKenzie, who set the stage for the adherence dialogue. The subsequent sessions explored topics such as the global impact of non-adherence, cross-continent perspectives, behavioral factors influencing adherence, and the role of technology and artificial intelligence in the physician–patient relationship. The Congress also emphasized the importance of the patient's voice in bridging relationships to improve medication adherence and concluded with reflections on key takeaways and future directions.

### The congress sessions

This article provides a comprehensive summary of the content covered in the six sessions of the Congress mentioned above, highlighting the key discussions, insights, and takeaways from each session.

#### Session 1: The silent epidemic: Unmasking the global impact of non-adherence

This session, presented by Rabia Khan, BSc, MPH, FFPH (Public Health), highlighted the critical yet often underestimated issue of medication non-adherence, which significantly impacts both individual patients and healthcare systems. In the US alone, it is estimated that medication non-adherence leads to approximately 125,000 deaths every year. [3]. Despite decades of research and intervention efforts, adherence rates have shown little improvement. Studies reveal that 4% to 31% of patients fail to fill their first prescription, while 18% to 34% of those who do, fail to fill the second. Over time, more than half of patients either discontinue their medications or fail to adhere to prescribed regimens [3]. This decline is evident at every stage: for every 100 prescriptions written, only 50% to 70% are filled, 48% to 66% are picked up, 25% to 30% are taken as prescribed, and 15% to 20% are refilled as directed [4].

It is a misconception to attribute non-adherence solely to the patient. Instead, non-adherence reflects a system-wide failure involving HCPs, policymakers, and authorities. Adherence plays a pivotal role in chronic disease management, contributing to the reduction of premature mortality and the prevention of avoidable hospitalizations, while mitigating healthcare costs. [3]. Addressing non-adherence is a critical strategy for reducing the strain on healthcare systems.

In the post-COVID-19 era, rising levels of mistrust and the spread of misinformation appear to have contributed to non-adherence. Findings from a global survey conducted in 2023 indicate that only 51% of respondents trust their healthcare system to deliver optimal care, with trust levels dropping to as low as 15% in certain countries [5]. Since trust in the healthcare system often shapes perceptions of individual healthcare providers, rebuilding institutional trust is crucial to fostering confidence in HCPs. Evidence demonstrates a strong correlation between trust in healthcare providers and improved adherence, highlighting the need to strengthen the relationship between patients and healthcare systems.

During this session, non-adherence was reframed as a system-wide challenge rather than being viewed solely as the responsibility of individual patients. Effective communication and strong patient-provider relationships are essential, framing adherence as a collective responsibility. Recognizing and addressing non-adherence as a systemic challenge is crucial for improving healthcare delivery and outcomes worldwide.

#### Session 2: Non-adherence without borders: Cross-continent perspectives and local realities

Nathorn Chaiyakunapruk, PharmD, PhD, (Health Economics), Assoc. Arintaya Phrommintikul, MD, (Cardiologist), and Miguel Angel Díaz Aguilera, MD (Integrated Medicine), addressed the global challenge of medication non-adherence and its substantial clinical and economic implications during this session. Policymakers require robust health economics data to grasp the impact of non-adherence and prioritize interventions fully. While clinical benefits of adherence are evident, quantifying cost savings for payer organizations serves as a powerful incentive for integrating adherence initiatives into national health policies; however, those must be quantified for each country to reflect local challenges and inform national decision-making.

The session drew attention to the burden of non-adherence presenting a cardiovascular disease (CVD) study. CVD remains the leading global cause of mortality, with a significant proportion of patients failing to achieve optimal risk factor control [6]. The reason is mainly linked to three risk factors: patient-related factors (notably non-adherence), healthcare provider-related factors, and healthcare system-related factors. Among these, low medication adherence is strongly associated with poor outcomes and increased costs. There is an inverse relationship between medication adherence and adverse cardiovascular outcomes; notably, a 20% improvement in adherence correlates with an 8% reduction in cardiovascular events and a 12% decrease in mortality [7].

Health economics data from Mexico, Thailand, and China demonstrated the potential benefits of improved CVD medication adherence. Current adherence rates stand at 50% in Mexico, 53% in Thailand, and 19% in China [8–10]. A simulation model projected that increasing adherence to optimal levels among 1,000 patients could prevent 42 cardiovascular events in Mexico, 34 in Thailand, and 63 in China, while yielding cost savings from a healthcare system perspective of $399, $290, and $552, per patient, respectively, in US dollars [11]. The total cost savings associated with achieving optimal adherence from a societal perspective were $412, $316, and $700 per patient, respectively. These findings emphasize that adherence improvements enhance clinical outcomes and yield cost savings from societal and healthcare system perspectives.

Despite advances in medical treatments and new drug development, chronic disease management has not improved primarily because of non-adherence to medication. Non-adherence in conditions such as diabetes, hypertension, and hyperlipidemia continue to drive avoidable complications, mortality, and healthcare costs [3]. This issue is rarely addressed in national health policy agendas, where interventions often overemphasize patient responsibility. However, systemic drivers, including patient-provider interactions, prescription refill processes, and out-of-pocket costs, are critical contributors. Innovative solutions, such as health apps, are emerging to empower patients, optimize treatment adherence, and improve disease monitoring. These tools represent a shift toward patient-centric care, bridging gaps in adherence by addressing both systemic and behavioral barriers. Improving adherence to currently available medications could achieve greater public health gains than developing new medical treatments.

The session emphasized the need to prioritize adherence within national healthcare agendas. Improving chronic disease outcomes and reducing healthcare cost burdens requires a collective effort involving policymakers, HCPs, payers, and the broader health community. Addressing adherence as a systemic issue is essential for fostering environments that support sustained and effective patient engagement in their treatments.

#### Session 3: “Don’t remind me to take my medication”: Exploring behaviors behind medication non-adherence

John Weinman, PhD, (Health Psychology) explored the complexities of medication adherence, revealing that it extends far beyond simple forgetfulness. While reminders may occasionally help, they are effective only for individuals already motivated to adhere. For patients who are non-adherent, reminders alone are insufficient. Research has identified over 700 factors influencing medication adherence, emphasizing the complex determinants of this behavior [12]. Addressing adherence effectively requires moving beyond conventional assumptions and understanding the behavioral drivers underlying this challenge [13].

Behavioral science offers valuable frameworks for understanding the nuances of adherence. One such model, the COM-B framework, classifies adherence determinants into three primary domains: capability, opportunity, and motivation [14]. These factors are patient-dependent, and often context-specific. For instance, a major illness diagnosis can profoundly impact a patient’s identity and sense of self, with treatment serving as a constant reminder of their condition. In response to this, some patients may avoid medication as an attempt to preserve their “healthy” self-image, while others may test the necessity of the treatment, reflecting complex psychological mechanisms [15].

To change adherence behaviors, it is critical to understand its root causes from an individual’s perspective. Behavioral diagnosis allows HCPs to identify specific barriers and design tailored interventions. The approach goes beyond merely instructing patients but instead, it emphasizes empathetic, non-judgmental conversations. Patients are often reluctant to discuss non-adherence, making open discussions essential for uncovering concerns, barriers, and motivations.

HCPs are encouraged to ask open-ended questions to understand the patient’s perspective, concerns, challenges, motivations, and willingness to change. HCPs must also ensure that patients clearly understand their treatment and why it is necessary. Through these collaborative discussions, HCPs can co-create practical plans with patients, addressing logistical details like when, where, and how medications should be taken in order to foster positive habits. Regular follow-ups are equally important since they provide ongoing support to help and maintain medication routines.

This session underscored that improving adherence requires integrating behavioral science into clinical practice. Raising awareness about non-adherence as a systemic problem and equipping HCPs with behavioral diagnostic tools can lead to more effective, patient-centered interventions. By understanding the behavioral drivers of adherence, HCPs can use behavioral skills to enable patients to engage more positively with their treatment, ultimately improving health outcomes and quality of life.

#### Session 4: Technology: AI and the physician–patient relationship

Ngiap Chuan Tan, MD (Family Medicine) and Evan Muse, MD, PhD (Cardiology) led a discussion on the transformative impact of artificial intelligence (AI) on healthcare delivery. The integration of AI into healthcare has shifted how medical care is delivered from traditional clinic- and hospital-based models to data-driven, technology-enabled approaches. The rise of telemedicine during the COVID-19 pandemic exemplified how technology can reshape healthcare services [16]. With the proliferation of health data and advancements in digital medicine, sensors now enable remote monitoring of various organ systems. AI leverages these data to enhance prevention and disease management, offering unprecedented opportunities for improving healthcare outcomes.

AI has advanced significantly throughout the years, evolving from feature engineering and deep learning to foundation models that power advanced large language models (LLMs). A recent study compared LLM chatbot responses to physicians, revealing that chatbots can provide high-quality insights, and are often perceived as more empathetic than physicians [17].

While AI’s capabilities inspire optimism, its increasing role raises concerns about its impact on the physician–patient relationship, a bond rooted in trust, honesty, and effective communication. For physicians, AI offers potential solutions to long-standing challenges. Administrative burdens during consultations often detract HCPs from time spent with patients, leading to dissatisfaction. AI can alleviate these repetitive tasks, allowing physicians to focus more on patient interaction and enhanced communication [18]. From the patient’s perspective, AI is met with both hope and apprehension. While it is recognized as a means to reduce wait times and streamline access to care, there are concerns about its deprivation of human elements in their healthcare experience [19].

However, AI’s limitations highlight a paradox in expectations [19]. While it enhances efficiency and enables physicians to see more patients, concerns persist about reduced emotional connection, de-skilling in observational abilities, and the absence of human touch. Patients value the empathy and emotional support inherent in human relationships—qualities AI cannot replicate at present. These challenges underscore the importance of integrating AI to preserve healthcare’s compassionate and interpersonal nature.

The future of AI in healthcare depends on the medical community’s ability to set standards that ensure AI systems are safe, unbiased, and equitable. Patients expect HCPs to address AI safety, data integrity, costs, and autonomy concerns. Physicians must balance embracing technological advancements and applying critical thinking to ensure AI is implemented responsibly. Rather than uncritically accepting or entirely rejecting AI, HCPs should develop a nuanced understanding of its capabilities and limitations, using it as a tool to complement, not replace, the human aspects of care. As highlighted by the ENABLE COST recommendations, addressing non-adherence to medications is a universal challenge that requires urgent attention [3, 20, 21], leveraging digital technologies to improve patient outcomes, foster collaboration, and enhance healthcare sustainability through investment, policy support, and data-driven strategies [22].

#### Session 5: Patient’s voice: Bridging the relationship to improve medication adherence

Ms. Heidi Floyd (Patient Advocate), delivered a moving testimonial during the session, sharing her unique experience in oncology. Heidi’s journey began during her college years as a caregiver for her mother, who battled breast cancer. At the time, cancer was surrounded by stigma and shame, making the journey isolating and emotionally challenging. Years later, while pregnant with her fourth child, Heidi faced her own breast cancer diagnosis. Despite her prior caregiving experience, she felt lost and fearful, realizing that every patient’s journey is unique. Her initial physician was unable to treat her due to her pregnancy, requiring her to seek a second opinion. This new physician established a trusting relationship with her by dedicating time to meaningful conversations, illustrating the foundational importance of communication in healthcare.

Reflecting on her experience, Ms. Floyd emphasized that every interaction between a patient and their HCPs, whether positive or negative, contributes to the overall patient experience. This experience extends beyond the physician to include every individual a patient encounters, from the receptionist to the nurse taking vitals. Each touchpoint shapes the patient’s perception of care, reinforcing the need for fostering connection throughout the journey.

Ms. Floyd also described the overwhelming emotional turmoil following a cancer diagnosis. Patients often fixate on the diagnosis itself, making it difficult to process subsequent information their physician provides. The immediate concerns about organizing their lives in the face of uncertainty can overshadow other critical details. Unlike her mother’s generation, she noted that today’s patients feel more empowered to ask questions and advocate for themselves. This boldness is vital, as the patient’s voice plays a crucial role in shaping their care. Patients must actively engage with their HCPs, expressing concerns, stress, and fears while confidently asking questions.

The concept of"quiet quitting,"was introduced referring to patients disengaging from treatment without informing their HCPs. This phenomenon highlights the importance of fostering an environment where patients feel their voices are valued. Strong, communicative relationships with HCPs can help prevent disengagement, allowing for collaborative problem-solving and the adaptation of adherence to care plans.

In this session, Ms. Floyd stressed the need for healthcare professionals to connect with patients from the beginning. Simple actions, such as asking thoughtful questions and demonstrating empathy, can help patients feel heard and valued. A welcoming and inclusive atmosphere enhances this relationship, with consistent discussions across various touchpoints in the patient care journey ensuring continuity. By showing patients that their voice matters and placing it at the center of their care experience, providers can empower them to speak up.

#### Session 6: Adherence: Whose problem is it?

Medication adherence is a shared responsibility that requires collective effort across all levels of the healthcare system. While patients must actively engage in their care, HCPs, medical societies, policymakers, and manufacturers are equally crucial in creating a supportive and equitable environment that fosters adherence. This final session featured a multidisciplinary discussion, with contributions from John Weinman, PhD, (Health Psychology), Ms. Heidi Floyd (Patient Advocate), Nathorn Chaiyakunapruk, PharmD, PhD, (Health Economy), and Shoaib Afzal, MD, PhD, DMSc as a representative of the European Atherosclerosis Society—EAS, to explore how each stakeholder can contribute to improving adherence.

### The patient's role

Patients must be empowered to speak openly, engage with their HCPs, and share their challenges. Effective communication requires encouragement and a safe, non-judgmental space for patients to express their concerns. It is essential to view patients as active partners in the healthcare process rather than passive care recipients. Patients should be involved from the earliest stages of research and development, as their insights are invaluable for designing practical, effective solutions. HCPs, students, policymakers, and researchers should engage with patients regularly, recognizing that patients are the ultimate focus of healthcare efforts. Nurses and allied health professionals also play a key role in maintaining continuous communication with patients and ensuring their perspectives are heard.

### The role of HCPs

HCPs must address adherence proactively, particularly when prescribing long-term therapies. Creating an environment that emphasizes trust, empathy, and transparency can improve adherence rates. Training in adherence-related conversations should be integral to medical education, from medical school curricula to continuing education programs for practicing clinicians.

### The role of medical societies and health authorities

Medical societies and health authorities are instrumental in developing evidence-based guidelines. However, adherence is often overlooked in these documents. Including adherence-focused recommendations, both within specialties and across disciplines, can raise awareness and influence policymakers and manufacturers. These guidelines should educate stakeholders and provide actionable strategies to improve adherence. From a policy perspective, education is a key lever for change. Medical societies can share existing research and develop communication modules for medical students and practitioners, equipping them with skills to foster supportive patient environments. Policymakers can leverage this evidence to implement system-wide reforms that prioritize adherence.

### Manufacturers'contributions

Manufacturers can support adherence through innovative solutions, such as integrating QR codes on medication packaging that link to educational resources or videos. These tools can help patients understand the importance of adherence and incorporate treatments into their daily routines. However, accessibility and equity must be prioritized to ensure that these solutions do not widen disparities in care.

### Economic Considerations

The economic impact of adherence should not be overlooked. Tools and interventions to improve adherence must be evaluated for clinical and economic outcomes. Demonstrating economic value can provide compelling evidence to policymakers and other stakeholders, incentivizing investment in adherence initiatives. Highlighting the shared economic benefits for healthcare systems and patients is a powerful motivator for systemic change.

The discussion emphasized that improving adherence requires a holistic approach, with every stakeholder playing an active role. By addressing adherence through patient empowerment, professional education, policy development, and equitable innovation, the healthcare system can move toward a future where adherence is no longer a barrier to optimal health outcomes.

## Conclusion

Medication adherence is a global challenge with profound implications for patient outcomes, healthcare systems, and society at large. The 2024 *a:care* Congress highlighted the burden of non-adherence and the urgency for action, offering potential solutions to change how it is conceptualized and managed. However, change will not occur spontaneously. We must continue to raise awareness about medication non-adherence while sustaining the beginnings of a coordinated change effort from all stakeholders, including patients, HCPs, policymakers, medical societies, and manufacturers.

The relationship between patients and the healthcare system lies at the core of addressing adherence. This relationship must be grounded in trust, open communication, transparency, and genuine connection. Every component of the healthcare ecosystem, from front-line HCPs to policymakers, has a role to play in cultivating this relationship. Improving adherence requires a multi-faceted approach. Technological innovations offer new opportunities to support adherence, while evidence-based guidelines that integrate adherence strategies can further enhance awareness and implementation. Ensuring accessibility and equity is paramount to prevent disparities and guarantee solutions benefit all patients. A paradigm shift is essential to make healthcare more sustainable. By aligning efforts across stakeholders, the healthcare system can build a future with higher treatment adherence rates, ensuring that adherence becomes a priority for global health initiatives.

## Data Availability

The replay of the congress is available online, on the a:care congress website (https://acarepro.abbott.com/acare-congress-2024-sessions/).

## Abbreviations

AI: Artificial intelligence
CVD: Cardiovascular disease
HCP: Health Care Providers
LLM: Large language models
Q&A: Questions and answers
US: United-States
WHO: World Health Organization
